# Editorial: Advances in crop biomass production based on multi-omics approach

**DOI:** 10.3389/fpls.2023.1155442

**Published:** 2023-04-19

**Authors:** Yin Li, Weizhen Liu, Xingtan Zhang, Shouchuang Wang, Ramin Yadegari, Jianping Wang

**Affiliations:** ^1^ The Genetic Engineering International Cooperation Base of Chinese Ministry of Science and Technology, The Key Laboratory of Molecular Biophysics of Chinese Ministry of Education, College of Life Science and Technology, Huazhong University of Science & Technology, Wuhan, China; ^2^ School of Computer Science and Artificial Intelligence, Wuhan University of Technology, Wuhan, China; ^3^ Shenzhen Branch, Guangdong Laboratory for Lingnan Modern Agriculture, Genome Analysis Laboratory of the Ministry of Agriculture, Agricultural Genomics Institute at Shenzhen, Chinese Academy of Agricultural Sciences, Shenzhen, China; ^4^ Hainan Yazhou Bay Seed Laboratory, Sanya Nanfan Research Institute of Hainan University, Sanya, China; ^5^ School of Plant Sciences, University of Arizona, Tucson, AZ, United States; ^6^ Agronomy Department, University of Florida, Gainesville, FL, United States

**Keywords:** genomics, multi-omics, biomass production, biomass related traits, bioinformatics, biomass and bio energy crops

## Introduction

While as the dominant source of energy during the past century, the detrimental impacts of fossil fuels have become apparent in environmental pollution, unsustainability, and global warming (Sharif et al., 2021). With increasing efforts and capitalization on renewable energy technologies, bioenergy has become one important type of renewable energy. Biomass of plants is an important feedstock of bioenergy production. Plants suitable for biomass production share common characteristics: high yield (of dry matter or a type of biomass, *i.e.*, starch or sugar), low agronomic inputs, and low nutrition requirements. Based on these features, woody species (*e.g.*, willow and poplar), grasses (*e.g.*, sugarcane, switchgrass, and *Miscanthus*), aquatic plants (*e.g.*, algae and duckweed), and oil plants have been considered biomass plants. Additionally, wheat and rice straw are important biomass sources. Biomass has several types according to the source species, the moisture content, and composition of biomass material, such as lignocellulosic biomass from woody plants, biomass from grasses (including cellulosic biomass from grasses or extracted starch/sugar), aquatic plant biomass, and manures (McKendry, 2002). In turn, these biomass types are compatible with different bio-conversion methods, *e.g.*, combustion, fermentation, gasification, pyrolysis, and mechanical extraction of starch or oils. Recently, numerous efforts have been made to convert biomass to high-value chemicals and bio-based materials (Anchan and Dutta, 2021).

Downstream utilizations of biomass (*e.g.*, conversion to biofuels or bio-based chemicals) requires multiple disciplines, such as agricultural science, microbiology, and chemistry. By contrast, upstream knowledge of biomass, such as the genetic determinants of biomass-related traits and molecular mechanisms of biomass accumulation and composition, relies on plant biology, and agricultural science. Notably, many biomass plants with large and complex genomes (such as sugarcane) have been less studied or have bottlenecks in transformation and traditional genetics (Zhang et al., 2018; Wang et al., 2021; An et al., 2021; Chen et al., 2022). Recently, research on biomass and bioenergy plants has been advanced rapidly due to the development of genomics. For example, state-of-the-art genomic technologies facilitated the successful assembly of reference genomes for sugarcane, *Miscanthus*, and switchgrass (Zhang et al., 2018; Mitros et al., 2020; Lovell et al., 2021). Though huge diversity within and among biomass crops provides invaluable resources for biomass utilization, understanding of biomass production mechanisms is still limited due to shortage of molecular and omic resources and challenges of functional studies. It has become apparent that synergistic integration of multiple omic technologies (*e.g.*, transcriptomics, proteomics, epigenomics, metabolomics, and phenomics) serves as a key approach to circumvent the challenges. This Research Topic includes seven research articles and two reviews, covering several biomass species, including maize, sorghum, sugarcane, rice, and oil plants to reveal the current advances of multi-omics in addressing the mechanisms of biomass production.

## Advances in multi-omic technologies and resources facilitate studies on biomass-related traits

This section showcases how omic technologies and resources can facilitate biomass studies. Voelker et al. reported the genome assemblies of 10 sorghum accessions including sweet and non-sweet sorghum genotypes (Boatwright et al.; Kumar et al., 2022). A large number of structural variations (SVs) were identified, which highlighted the SV-related functional difference between sweet and non-sweet sorghum genotypes. Wang et al. developed an image-based phenotypic acquisition method to characterize leaf-sheath traits in detail and applied the method to genome-wide association studies (GWAS), providing a detailed genetic architecture of leaf-sheath morphology. Guo et al. presented an integrative genomic database for oil plants, the Genomic Information Repository for Oil Plants (GROP, www.grop.site), which hosts 22 reference genomes of 18 species with 46 transcriptome datasets (Bayer et al., 2017; Unver et al., 2017; Wang et al., 2018; Song et al., 2020; Sturtevant et al., 2020; Chen et al., 2021). The construction of such an omics repository addresses the need to integrate, share, and analyze the omics data across oil plants for the research community. In addition, Tu et al. reviewed the major applications of regular short-read RNA-seq in plant biology, described a cohort of representative RNA-seq-analysis tools in model plants and major crops, and emphasized that the full utilization of fruitful RNA-seq resources will promote the omic research on under studied species (including biomass crops) to a high level.

## Applications of omic approaches provide insights into biomass-related biology

This section collects representative papers using omic technologies to gain insights into biomass-related biological questions. Sugarcane is one of the key biomass and bioenergy crops, providing about 80% of global sugar production and 40% of ethanol production (Zhang et al., 2018). Efforts have been made to investigate the molecular mechanisms of sugar accumulation in sugarcane and in the comparable species sweet sorghum (Li et al., 2018; Li et al., 2019a; Li et al., 2019b), from sugar transportation and physiology to transcriptome and quantitative trait loci mapping (Babu et al., 2009; Liu et al.; Moore, 2005; Aitken et al., 2006; Casu et al., 2007; Zhang et al., 2021). Yuan et al. performed transcriptomic and metabolomic studies on two sugarcane varieties and revealed candidate genes for sucrose metabolism, stem texture, and rind color. While the genes associated with stem sugar accumulation have been identified in sugarcane (Casu et al., 2007; Zhang et al., 2021), epigenetic regulation remains elusive. Xue et al. profiled the DNA methylation in sugarcane (*Saccharum officinarum*) leaves, roots, rinds, and piths, and observed DNA methylation valleys (DMVs) overlapped with transcription factors and sucrose-related genes, indicating the involvement of epigenetic regulation in sucrose metabolism. Liu et al. revealed the link of OsPRR37, a key component of the rice circadian clock, with biomass production through DNA methylation analysis. Overexpression of OsPRR37 in rice led to suppressed growth and lowered biomass likely through the diurnal changes of DNA methylation regulators (such as ROS1A/DNG702) to hypo-methylate a key signal component controlling metabolism, OsHXK1 (Zheng et al., 2021; Zhou et al., 2021). Ain et al. presented a comprehensive review on recent progress in the identification of molecular and genetic factors regulating growth, biomass accumulation, and assimilate partitioning in bioenergy crops. The review highlights a plethora of genes related to cell cycle, cell wall, hormones, and related transcription factors as the targets to improve photosynthesis, carbohydrate allocation, and biomass production in the bioenergy crops. Additionally, this topic also hosts an example of omics-enabled trait association study. Specifically, Wang et al. used comparative RNA-seq to profile seed-specific long-lived mRNA and identify a number of the long-lived mRNA associated with rice seed longevity.

## Concluding remarks

This Research Topic exemplifies that multi-omics represent an important route to strengthen the studies of biomass crops, particularly with complex genomes. Importantly, trends emerged from these articles that a combination of multiple omic resources and tools is a powerful approach to gaining new insights into biomass production and related traits. The discoveries will pave the road toward molecular design and breeding biomass crops with tailored bioenergy purposes.

## Author contributions

YL, WL, XZ, SW, RY, and JW drafted and revised this editorial based on this Research Topic’s contributions. All authors approved the submitted version.
